# Knowledge and Adherence to the National Guidelines for Malaria Case Management in Pregnancy among Healthcare Providers and Drug Outlet Dispensers in Rural, Western Kenya

**DOI:** 10.1371/journal.pone.0145616

**Published:** 2016-01-20

**Authors:** Christina Riley, Stephanie Dellicour, Peter Ouma, Urbanus Kioko, Feiko O. ter Kuile, Ahmeddin Omar, Simon Kariuki, Ann M. Buff, Meghna Desai, Julie Gutman

**Affiliations:** 1 Rollins School of Public Health, Emory University, Atlanta, United States of America; 2 Liverpool School of Tropical Medicine, Liverpool, United Kingdom; 3 KEMRI, Centre for Global Health Research, Kisumu, Kenya; 4 Malaria Control Unit, Ministry of Health, Nairobi, Kenya; 5 Malaria Branch, Division of Parasitic Diseases and Malaria, Center for Global Health, US Centers for Disease Control and Prevention, Atlanta, United States of America; 6 US President’s Malaria Initiative, Nairobi, Kenya; 7 Centers for Disease Control and Prevention, Kisumu, Kenya; Centro de Pesquisa Rene Rachou/Fundação Oswaldo Cruz (Fiocruz-Minas), BRAZIL

## Abstract

**Background:**

Although prompt, effective treatment is a cornerstone of malaria control, information on provider adherence to malaria in pregnancy (MIP) treatment guidelines is limited. Incorrect or sub-optimal treatment can adversely affect the mother and fetus. This study assessed provider knowledge of and adherence to national case management guidelines for uncomplicated MIP.

**Methods:**

We conducted a cross-sectional study from September to November 2013, in 51 health facilities (HF) and a randomly-selected sample of 39 drug outlets (DO) in the KEMRI/CDC Health and Demographic Surveillance System area in western Kenya. Provider knowledge of national treatment guidelines was assessed with standardized questionnaires. Correct practice required adequate diagnosis, pregnancy assessment, and treatment with correct drug and dosage. In HF, we conducted exit interviews in all women of childbearing age assessed for fever. In DO, simulated clients posing as first trimester pregnant women or as relatives of third trimester pregnant women collected standardized information.

**Results:**

Correct MIP case management knowledge and practice were observed in 45% and 31% of HF and 0% and 3% of DO encounters, respectively. The correct drug and dosage for pregnancy trimester was prescribed in 62% of HF and 42% of DO encounters; correct prescription occurred less often in first than in second/ third trimesters (HF: 24% vs. 65%, p<0.01; DO: 0% vs. 40%, p<0.01). Sulfadoxine-pyrimethamine, which is not recommended for malaria treatment, was prescribed in 3% of HF and 18% of DO encounters. Exposure to artemether-lumefantrine in first trimester, which is contraindicated, occurred in 29% and 49% of HF and DO encounters, respectively.

**Conclusion:**

This study highlights knowledge inadequacies and incorrect prescribing practices in the treatment of MIP. Particularly concerning is the prescription of contraindicated medications in the first trimester. These issues should be addressed through comprehensive trainings and increased supportive supervision. Additional innovative means to improve care should be explored.

## Introduction

Approximately 125 million pregnancies occur in areas at risk of *Plasmodium falciparum* and *P*. *vivax* infections every year; an estimated 1.3 million of these occur in Kenya [1]. Malaria in pregnancy (MiP) can have devastating consequences for the woman and her unborn baby, including maternal anemia, fetal loss, intrauterine growth retardation, premature delivery and low birth weight (LBW) with increased risk for neonatal death [2]. In line with World Health Organization (WHO) recommendations, the Kenya Ministry of Health (MoH) recommends that pregnant women use long-lasting insecticidal nets (LLINs), intermittent preventive treatment in pregnancy (IPTp) with sulfadoxine-pyrimethamine (SP), and receive prompt and effective diagnosis and treatment of malaria with a safe drug in order to prevent adverse consequences.

The Kenya National Treatment Guidelines recommend artemether-lumefantrine (AL), an artemisinin combination therapy (ACT), as the first-line treatment for uncomplicated *P*. *falciparum* malaria in the general population and women in second and third trimesters of pregnancy. However, due to insufficient safety data on ACTs [3–5], AL is contraindicated in first trimester, and oral quinine is recommended instead [6–8]. In practice, all women of child-bearing age (WOCBA) must be assessed for pregnancy and gestational age. Since 2010, Kenya Ministry of Health (MoH) treatment guidelines recommend universal parasitological diagnosis. Antimalarial treatment on the basis of clinical suspicion of malaria should only be considered in situations where a parasitological diagnosis is not accessible, particularly in vulnerable populations such as pregnant women [6]. We found that 77% of WOCBA and pregnant women in health facilities (HFs) and 5% in drug outlets (DOs) in Kenya received parasitologically confirmed diagnosis [9].

Limited data exists on healthcare provider adherence to case management guidelines for MiP. Recent studies from Kenya have reported high levels (80–90%) of adherence to malaria treatment guidelines for the general population [10–12]. However, a recent systematic review and meta-analysis of global MiP case management reported that overall, healthcare providers followed the pregnancy specific treatment guidelines for malaria in 28% of first trimester encounters and 72% of second and third trimester encounters [13]. Inadvertent exposure to ACTs in the first trimester and continued use of ineffective treatment regimens, such as SP, has been observed in a number of countries; very few providers know that ACTs are potentially teratogenic (harmful to foetal development) [13–18]. In Uganda, 70% of women with a first-trimester pregnancy received a contraindicated antimalarial and less than 6% received quinine, the drug recommended for treatment of malaria in the first trimester of pregnancy [18]. In Tanzania, 43% of drug dispensers in registered pharmacies offered AL regardless of the pregnant client’s gestational age; only 20% knew that AL was contraindicated during the first trimester [15].

Understanding provider prescribing behaviour in pregnant women is essential to ensure adequate case management and to minimize potential harmful exposures; this is relevant in health facilities and drug outlets because each comprise a significant proportion of healthcare service utilization [19]. About 25% of the total population are WOCBA and up to 14% could be pregnant at any time; therefore, it is crucial that providers recognize potentially teratogenic medications and assess WOCBA for pregnancy status and gestational age. This cross-sectional study assessed healthcare provider and drug dispenser prescribing behaviors and knowledge of malaria treatment guidelines for pregnant clients in a malaria-endemic region of western Kenya.

## Methods

Knowledge of MiP guidelines and self-reported prescribing behavior for MiP case management was assessed by structured provider questionnaires administered to healthcare providers and drug dispensers. Adherence to the National Malaria Treatment Guidelines (assessed via prescribing practice) was observed by a) exit interviews with WOCBA (18–49 years) and pregnant clients being treated for febrile illness at all HFs within the study area, and b) use of a simulated-client approach within randomly sampled DOs. Provider knowledge questionnaires were administered following completion of the provider practice component to avoid influencing provider behavior.

### Study Site & Sampling

The study was conducted from September to November 2013, in rural Siaya County in western Kenya. The study area included the Kenya Medical Research Institute (KEMRI) and U.S. Centers for Disease Control and Prevention (CDC) Public Health Collaboration Health and Demographic Surveillance System (HDSS)[20] as well as a 5 km radius surrounding the HDSS. Malaria transmission is perennial and holo-endemic. In this area, approximately 20% of pregnant women are parasitemic at first antenatal clinic visit [21], and 18% of women delivering in Siaya District Hospital had placental malaria [22].

#### Health facility selection

All HFs in the HDSS area and within a 5km radius surrounding the HDSS area were assessed for eligibility. Facilities were eligible if they provided outpatient care and the facility in-charge consented to participate. Facilities with ongoing studies that could have influenced the study results were excluded.

#### Drug outlets selection

Prior to the start of data collection, a census was conducted of all registered and unregistered (i.e., informal) DOs selling antimalarial drugs within the HDSS border [23]. DOs were eligible if they sold antimalarials and consented to participate. All home-based shops were excluded given that provider practice was assessed via simulated client which was not feasible in this setting. This sample size allowed estimation of the proportion of providers (one per facility) with adequate knowledge with 14% precision at 80% power, assuming that 45% of providers have adequate knowledge and prescribing practice [15].

### Data Collection

#### Provider surveys in health facilities & drug outlets

A fieldworker administered the structured knowledge questionnaire to one to two providers per HF (dependent upon number of staff treating WOCBA and pregnant women) and one provider per DO to assess knowledge and self-reported prescribing practice. Providers were selected from all providers who treated WOCBA for malaria and were available at the time of interview. These interviews were carried out immediately following the completion of exit interviews and one week after client simulations (described below) by a different fieldworker.

#### Exit interviews in health facilities

All women who completed a provider consultation in either the outpatient department (OPD) or antenatal care (ANC) clinic were approached after receiving all prescribed medications by fieldworkers trained for 10 days on proper interviewing techniques. Fieldworkers assessed eligibility and obtained informed consent from women presenting with febrile illness. Facilities were visited for a minimum of one day and a maximum of 10 days. Field workers remained at a facility for consecutive days either until they had interviewed at least one woman in each of the following categories per facility: 1) WOCBA who could potentially be pregnant, 2) early pregnancy (first trimester, defined as up to 14 weeks inclusive), and 3) late pregnancy (second and third trimesters, defined as 15 weeks or greater), or after 10 consecutive clinic days. Exit interviews were conducted following a structured questionnaire. Pregnancy status was based on patient report; gestational age and trimester were calculated from date of last menstrual period (LMP). In encounters where an antimalarial contraindicated in pregnancy had been prescribed, the patient was given the recommended treatment by the study clinician and instructed not to take the incorrect medication. Fieldworkers were positioned outside of the clinic and the interactions could not be overheard or observed by clinic staff; health providers were not aware of changes to medications until after completion of the study.

#### Simulated clients in drug outlets

The simulated-client (i.e., mystery clients [24,25]) approach was used to assess prescribing practice within DOs. Three scenarios (WOCBA, early pregnancy, late pregnancy) were simulated at each outlet. Female fieldworkers presented as either WOCBA or in early pregnancy, and male fieldworkers presented as the husband of a WOCBA or woman in third-trimester pregnancy. A single provider-client simulation had the potential to include up to two scenarios: non-pregnant and pregnant. The simulated clients were trained not to disclose pregnancy status unless it was asked by the dispenser. If dispensers failed to assess pregnancy status, the simulated clients would disclose pregnancy status after being advised on a treatment regimen; this pregnancy scenario was then assessed based on changes in practice, or lack thereof, made after pregnancy disclosure. The checklist for the simulated-client encounter was completed immediately after each interaction.

### Definitions

Correct practice and adequate knowledge definitions (Table 1) were based on the 2010 Kenya National Malaria Treatment Guidelines (MTGs) [7] and the 2010 WHO Malaria Treatment Guidelines [6]. Correct case management required adequate diagnosis (additional information presented in [9]), pregnancy assessment, and treatment with correct drug and dosage. For the knowledge assessment, providers were asked to name the recommended first line therapy, while for exit interviews and client simulations (practice component), either first- or second-line medications were considered correct.

**Table 1:** Definitions of Correct Practice & Adequate Knowledge.

**Correct Malaria Diagnosis**	
Utilization of microscopy or rapid diagnostic test	
Clinical diagnosis when diagnostic test unavailable	
**Correct Pregnancy Assessment**	
Inquired about pregnancy and/ or offered pregnancy test	
Inquired about LMP, gestational age, or measured fundal height or palpated	
**Correct Treatment & Dosage**	
*Acceptable Knowledge Answers*	*Acceptable Prescriptions in Practice*
***Non-pregnant***	***Non-pregnant***
1st-line: Artemether-lumefantrine	Artemether-lumefantrine
2nd-line: DHA-piperaquine	DHA-piperaquine
	Quinine
***1st Trimester***	***1st Trimester***
Quinine	Quinine
***2nd/3rd Trimester***	***2nd/3rd Trimester***
Quinine	Quinine
Artemether-lumefantrine	Artemether-lumefantrine
	DHA-piperaquine
**Treatment regimens**:	
Artemether-lumefantrine tablets (20/120 mg): 4 tablets, 2 times daily for 3 days (4x2x3)	
DHA-piperaquine tablets (40/320 mg): 3 or 4 tablets, once daily for 3 days (3x1x3) or (4x1x3)	
Quinine: 2 tablets of 300 mg, 3 times daily for 7 days (2x3x7)*	

Acronyms: LMP, date of last menstruation period; DHA, dihydroartemisinin,

*Differs from standard adult dosing regimen of 5 full days

### Data Management & Statistical Analysis

Data from the provider surveys were collected via personal digital assistant (PDA), and data from simulated clients and exit interviews were collected via scannable forms. We used SAS 9.3 (SAS Institute, Cary, NC, USA) for analysis.

Chi-square test or Fisher exact test were used to assess statistical significance (p≤0.05) of comparisons between categorical variables. We calculated the proportion of providers, accounting for clustering at the facility level, who adequately assessed for pregnancy and correctly prescribed treatment (correct treatment was defined as prescribing the correct medication and dosage); these measures were used to define overall correct MiP case management practice. Logistic regressions were performed at the individual-provider level, accounting for clustering at the facility level to identify significant (p≤0.05) predictors of case management knowledge. Intra-cluster correlation at the facility level was accounted for in all analysis.

### Ethics

The study was approved by the Kenya Medical Research Institute (KEMRI) Ethical Review Committee and the institutional review boards of the Centers for Disease Control and Prevention, Liverpool School of Tropical Medicine, and Emory University. Written informed consent was obtained from all providers and patients prior to interviews. the component of the study assessing prescribing behaviour from drug outlets which involved simulated clients, verbal consent was sought from all drug outlet dispensers during the drug-outlet mapping for future participation in a study for assessment MiP treatment using simulated clients. It was not possible to obtain informed consent at the time of the simulated client interaction, as it was critical to the study design that the provider was unaware that the client was assessing practice. No personal information on the drug dispenser was collected during the simulated client interview, thus verbal consent (documented in the drug-outlet mapping case report forms) was considered sufficient and was approved by all ethical review boards.

## Results

### Provider Knowledge of National Malaria Treatment Guidelines

We surveyed 112 providers across 86 facilities; 75 in HFs and 37 in DOs. Of respondents, 44% were nursing staff, 16% were clinical officers or physicians, 18% pharmacists, and 13% were shopkeepers. Sixty-nine percent of providers stated that they both prescribed and dispensed medication (Table 2).

**Table 2:** Provider characteristics from the provider survey on national malaria treatment guidelines.

	Overall		Health facilities		Drug Outlets	
Provider/Dispenser Characteristics	N	%	N	%	N	%
	112		75		37	
**Sex**						
Male	54	48.2	39	52.0	15	40.5
Female	58	51.8	36	48.0	22	59.5
**Respondent Cadre**						
Registered Nurse	33	29.5	32	42.7	1	2.7
Enrolled Nurse	16	14.3	16	21.3	0	0.0
Clinical Officer/Physician	18	16.1	17	22.7	1	2.7
Pharmacist	20	17.9	5	6.7	15	40.5
Shopkeeper	15	13.4	0	0.0	15	40.5
CHW/VR/other*	10	8.9	5	6.7	5	13.5
**Professional Qualification**						
Primary School	9	8.0	2	2.7	7	18.9
Secondary School	23	20.5	7	9.3	16	43.2
Higher Education	19	17.0	13	17.3	6	16.2
Clinical Officer/MD	14	12.5	13	17.3	1	2.7
Registered Midwife/Nurse	22	19.6	20	26.7	2	5.4
Enrolled Midwife/Nurse	11	9.8	10	13.3	1	2.7
Pharmacist	4	3.6	1	1.3	3	8.1
Other technical	10	8.9	9	12.0	1	2.7

* CHW—community health worker, VR = village reporter, ‘other’ included clerk, economist, statistical clerk, and support staff

Overall, 75% (84/112) of providers were aware of the National Malaria Treatment Guidelines (MTGs); knowledge was higher among HF than DO providers (S1 Table). Eighty-eight percent (99/112) of providers reported ‘always’ or ‘sometimes’ performing a pregnancy assessment; 79% (78/99) of whom reported asking for LMP, and 48% reported offering a pregnancy test (48/99).

Forty-seven percent (35/75) of HF providers knew the correct first-line treatment and dosage for all pregnancy scenarios compared to none (0%) of the 37 drug dispensers. Over half (56%) of HF providers and no drug dispensers knew the correct treatment for first trimester patients (p<0.01); 85% (64/75) of HF and 41% (15/37) of drug outlet providers knew the correct treatment for second and third trimester patients (p<0.01). Provider knowledge was considerably higher for first-line treatment versus second-line treatment (82% v 16%, p<0.01) (Table 3).

**Table 3:** Adequate Provider Knowledge of Malaria in Pregnancy based on National malaria treatment guidelines comparing health facilities to drug outlets.

	Overall			Health Facilities			Drug Outlets			P-value*
	n = 112	%	95% CI	n = 75	%	95% CI	n = 37	%	95% CI	
**Consequences of MiP**	110	98.2	(95.7, 100.0)	74	98.7	(96.0, 100.0)	36	97.3	(92.0, 100.0)	0.61
**Awareness of MTGs**	84	75.0	(66.1, 83.9)	74	98.7	(96.0, 100.0)	10	27.0	(12.4, 41.6)	<0.01
**Pregnancy Assessment**	88	78.6	(70.7, 86.4)	70	93.3	(87.9, 98.8)	18	48.6	(32.2, 65.1)	<0.01
**Treatment & Dosage**	35	31.3	(22.0, 40.5)	35	46.7	(34.4, 59.0)	0	0.0		<0.01
**NP-1st Line**	92	82.1	(74.9, 89.4)	69	92.0	(86.1, 97.9)	23	62.2	(46.2, 78.1)	<0.01
NP-2nd Line	18	16.1	(8.9, 23.2)	15	20.0	(10.6, 29.4)	3	8.1	(0.0, 17.1)	<0.01
**1st Tri- 1st Line**	**42**	**37.5**	**(27.6, 47.4)**	**42**	**56.0**	**(43.6, 68.4)**	**0**	**0.0**		<0.01
**2nd/3rd Tri- 1st Line**	**79**	**70.5**	**(61.7, 79.3)**	**64**	**85.3**	**(77.1, 93.6)**	**15**	**40.5**	**(24.4, 56.7)**	<0.01
Severe MiP	69	61.6	(52.1, 71.1)	61	81.3	(71.8, 90.9)	8	21.6	(8.1, 35.2)	<0.01
**Adequate Knowledge**	**34**	**30.4**	**(21.3, 39.4)**	**34**	**45.3**	**(33.2, 57.5)**	**0**	**0.0**		<0.01

*Fisher Exact used for strata with <5 observations

Acronyms: MTG, malaria treatment guidelines, MiP, malaria in pregnancy, NP, non-pregnant; Tri, trimester of pregnancy

SP was incorrectly cited as the appropriate treatment for acute malaria in the following scenarios: a) first- and/or second-line treatment for adults by 3% of HF and 10% of DO providers, b) in first trimester pregnant patients by 9% and 39% of providers, c) in second and third trimester by 1% and 18% of providers, and d) for treatment of severe malaria in pregnancy by 3% and 10% of providers respectively. Two-thirds cited IPTp with SP as a preventive measure for MiP; however, only 67% of HF and 32% of DO providers knew that SP could be used only as preventive therapy and not as treatment. Additionally, 4% of HF and 10% of DO providers thought AL could be used as preventive therapy and another 41% of DO providers were unable to cite a drug for preventive treatment. An ACT was incorrectly cited as the appropriate treatment in first-trimester pregnancies by 5% of HF providers and 18% of drug-outlet providers; 56% of providers (71% and 27%, respectively) were aware that ACTs are contraindicated in first trimester (S2 Table).

#### Predictors of MiP case management knowledge

Significant predictors of adequate knowledge of MiP case management in the adjusted model included facility type and malaria case management training (Table 4). HF providers were more likely to possess adequate knowledge of correct case management than their DO counterparts (adjusted OR (aOR) = 2.8; 95% CI [0.9–8.4]) although this was not statistically significant. Having attended malaria case management training increased the likelihood of having adequate knowledge (aOR = 3.6; 95% CI [1.3–9.7]). Other factors including age, gender, respondent cadre, and education were also analysed but were not significantly associated with provider knowledge.

**Table 4:** Provider Characteristic Predictors of Adequate Knowledge of Malaria in Pregnancy Case-Management.

Provider Characteristic	N	%	Crude OR	95% CI	P	Adjusted OR*	95% CI	P
Facility Type**	**112**							
Health Facilities	**75**	67.0	4.3	(1.6, 11.7)	<0.01	2.8	(0.9, 8.4)	0.07
Drug Outlets (reference)	**37**	33.0	—	—	—	—	—	—
**Malaria Management Training**								
None	51	45.5	—	—	—	—	—	—
Yes	61	54.5	4.8	(1.9, 12.1)	<0.01	3.6	(1.3, 9.7)	0.01
**Sources of Information (reference = 'No')**								
CME as a source of info	63	56.3	1.5	(1.8, 10.4)	<0.01	—	—	—
**Knowledge Variable (reference = 'Not Known')**								
1st Trimester as Contraindication	63	56.3	6.1	(2.1, 17.8)	<0.01	—	—	—
Return to Facility if no Improvement	58	51.8	2.7	(1.2, 5.9)	0.02	—	—	—
Sleep under ITN	103	92.0	3.7	(0.9, 14.8)	0.07	—	—	—
SP can only be used for MiP Prevention	62	55.4	2.6	(1.0, 6.5)	0.05	—	—	—

*Adjusted model included facility type and malaria diagnostic training. CME & Knowledge variables could not be included in the multivariate model as the model did not converge when they were included.

**Facility type is stratified at the health facility versus drug outlet level; the fully stratified model was unstable due to quasi-complete separation of data points.

### MiP Case Management Practice: Exit Interviews in Health Facilities

After excluding 9 HFs due to ongoing studies that could have influenced study results, 52 HFs were eligible for the study; supervisors in 51 facilities (including four hospitals, 19 health centers, and 28 dispensaries) consented to participate (S3 Table). A total of 208 eligible patients were interviewed across participating HFs: 111 non-pregnant women, 21 women in the first trimester of pregnancy, and 76 women in the second and third trimesters (S4 Table).

#### Pregnancy assessment

Only 51% (107/208) of all patients were assessed for pregnancy status; 63% (61/97) of pregnant women were asked for their LMP (Table 5). A pregnancy test was offered to only 9% (19/208) women; offering a pregnancy test was significantly associated with first trimester of pregnancy, as might be expected, given that later in pregnancy, the status becomes more obvious on examination. Study fieldworkers identified 23 women who reported an LMP of greater than six weeks; none of whom were assessed for pregnancy status by the provider.

**Table 5:** Pregnancy assessment practice in health facilities as observed through exit interviews stratified across pregnancy status.

	Overall				Non-Pregnant		1st Trimester			2nd/3rd Trimester			
	N	%	95% CI	N	%	95% CI	N	%	95% CI	N	%	95% CI	P-value
***All Patients***	208			111			21			76			
Pregnancy Status Inquiry	91	43.8	(35.3, 52.2)	29	26.1	(16.9, 35.4)	16	76.2	(55.2, 97.2)	46	60.5	(49.2, 71.8)	<0.01
Pregnancy Test Offered	19	9.1	(5.1, 13.1)	4	3.6	(0.3, 6.9)	8	38.1	(16.4, 59.8)	7	9.2	(2.3, 16.1)	<0.01
LMP Inquiry	88	42.3	(33.4, 51.4)	27	24.3	(14.6, 34.0)	15	71.4	(49.7, 93.2)	46	60.5	(49.9, 71.2)	<0.01
Pregnancy Duration/Timing	66	68.0	(56.5, 79.6)	NA			13	61.9	(38.0, 85.8)	53	69.7	(58.1, 81.4)	0.5
Additional Confirmation*	41	42.3	(30.9, 53.6)	NA			5	23.8	(4.0, 43.6)	36	47.4	(35.2, 59.6)	0.06
**Correct pregnancy assessment**	**107**	**51.4**	**(41.9, 61.0)**	**27**	**24.3**	**(14.6, 34.0)**	**17**	**81.0**	**(60.9, 100.0)**	**63**	**82.9**	**(75.0, 90.8)**	**<0.01**

* Additional confirmation included palpation in first trimester, and palpation or observation in second/third trimester encounters.

#### Treatment prescribed

An antimalarial medication was prescribed to 98% (204/208) of women; the most frequent antimalarials prescribed were AL (83%), followed by quinine (14%), then SP (3%). Sixty-two percent (129/208) of women received the correct treatment (Table 6). Despite being contraindicated in first trimester, AL was prescribed in first trimester to six of 21 (29%) women. While the majority (73%, 125/172) of prescriptions for AL were for the correct dosage, the correct dose of quinine was prescribed only 29% (8/28) of the time. In all 20 incorrect-quinine-dosage encounters, this resulted in sub-therapeutic dosing, and in 70% (14/20) of these encounters, patients were prescribed less than the standard 5-day adult course of quinine. Due to the fact that AL was frequently prescribed irrespective of trimester, the correct drug and dosage was prescribed more frequently to non-pregnant patients (68%) and those in the second and third trimester of pregnancy (63%) than to those in first trimester (24%, p = 0.001). Correct case management was observed in only 31% (65/208) of patients, with no significant difference across HF types (S5 Table).

**Table 6:** Malaria treatment practice in health facilities as observed through exit interviews stratified across pregnancy status.

	Overall			Non-Pregnant			1st Trimester			2nd/3rd Trimester			
	N	%	95% CI	N	%	95% CI	N	%	95% CI	N	%	95% CI	P-value
	208			111			21			76			
**Prescribed Antimalarials**	204	**98.1**		**110**	**99.1**		**20**	**95.5**		**74**	**97.4**		
Proper Dosage (tabs x doses x days)*					**0.00**			**0.0**					
Artemether-lumefantrine	172	82.7	(77.5, 87.9)	105	94.6	(90.2, 98.9)	**6**	28.6	(4.0, 53.2)	61	80.3	(70.8, 89.8)	<0.01
*(4x2x3)*	125	72.7	(61.8, 82.6)	74	70.5	(55.4, 85.6)	**5**	83.3	(59.6, 100.0)	46	75.4	(58.4, 92.4)	<0.01
DHA-Piperaquine	2	1.0	(0.0, 2.3)	2	1.8	(0.0, 4.5)	0	0.0		0	0.0		
*(3x1x3)*	1	50.0	(0.0, 1.5)	1	50.0	(0.0, 2.8)		0.0			0.0		
Quinine	28	13.5	(9.1, 17.8)	4	3.6	(0.0, 8.1)	12	57.1	(33.6, 80.7)	12	15.8	(7.0, 24.6)	<0.01
*(2x3x7)*	7	25.0	(9.2, 40.8)	0	0.0		5	41.7	(11.6, 71.7)	2	16.7	(0.0, 39.8)	<0.01
*(150mgxN)*	1	3.6	(0.0, 11.0)	1	25.0	(0.0, 74.4)	0	0.0		0	0.0		
Sulfadoxine Pyrimethamine	7	3.4	(0.4, 6.4)	**1**	0.9	(0.0, 2.8)	**2**	9.5	(0.0, 22.0)	**4**	5.3	(0.0, 11.3)	0.06
*(3x1x1)*	5	71.4	(46.8, 96.0)	**0**	0.0		**2**	100.0	(0.0, 100.0)	**3**	75.0	(50.0, 100.0)	0.42
Artemether Injection	1	0.5	(0.0, 1.5)	1	0.9	(0.0, 2.8)	0	0.0		0	0.0		
*(60mg)*	1	100.0	(0.0, 100.0)	1	100.0	(0.0, 100.0)		0.0			0.0		
**Correct Drug**	194	93.3		111	100.0		12	57.1		74	97.4		
Correct Drug & Dosage	129	62.0	(52.2, 71.8)	**76**	68.5	(57.2, 79.7)	**5**	23.8	(5.3, 42.3)	**48**	63.2	(49.6, 76.7)	<0.01
**Concomitant Medications**					0.0								
Analgesic	147	70.7	(61.2, 80.2)	79	71.2	(59.3, 83.0)	16	76.2	(57.7, 94.7)	52	68.4	(55.7, 81.1)	0.78
Antibiotic	74	35.6	(26.5, 44.7)	37	33.3	(21.9, 44.8)	6	28.6	(10.2, 47.0)	31	40.8	(28.9, 52.6)	0.4
**1**^**st**^ **Antimalarial dose Directly Observed**	50	24.0	(14.4, 33.4)	25	22.5	(10.2, 34.8)	4	19.0	(2.9, 33.5)	21	27.6	(15.4, 39.9)	0.59
**Treatment Advice**					0.0								
Reason for Prescription	58	27.9	(20.7, 36.1)	32	28.8	(20.9, 38.3)	5	23.8	(5.3, 44.3)	21	27.6	(16.6, 38.7)	0.88
Side Effects	14	6.7	(3.1, 10.8)	5	4.5	(0.5, 8.8)	4	19.0	(1.2, 40.8)	5	6.6	(1.2, 12.3)	0.03
Any other advice**	45	21.6	(15.0, 28.3)	21	18.9	(11.0, 26.8)	5	23.8	(2.8, 44.8)	19	25.0	(14.0, 36.0)	0.63
**Any treatment Advice**	**82**	39.4	(31.2, 47.6)	**42**	37.8	(27.5, 48.2)	**10**	47.6	(24.5, 70.8)	**30**	39.5	(27.0, 52.0)	0.72

*The denominator for percentage for correct dosage is based on the number of patients receiving the specific antimalarial.

**Any other advice includes patient-reported advice by the provider including emphasis of complete medication regimen, eating prior to taking medication, sleeping under ITNs, etc.

### MiP Case Management Practice: Simulated Clients in Drug Outlets

**Fig 1:**
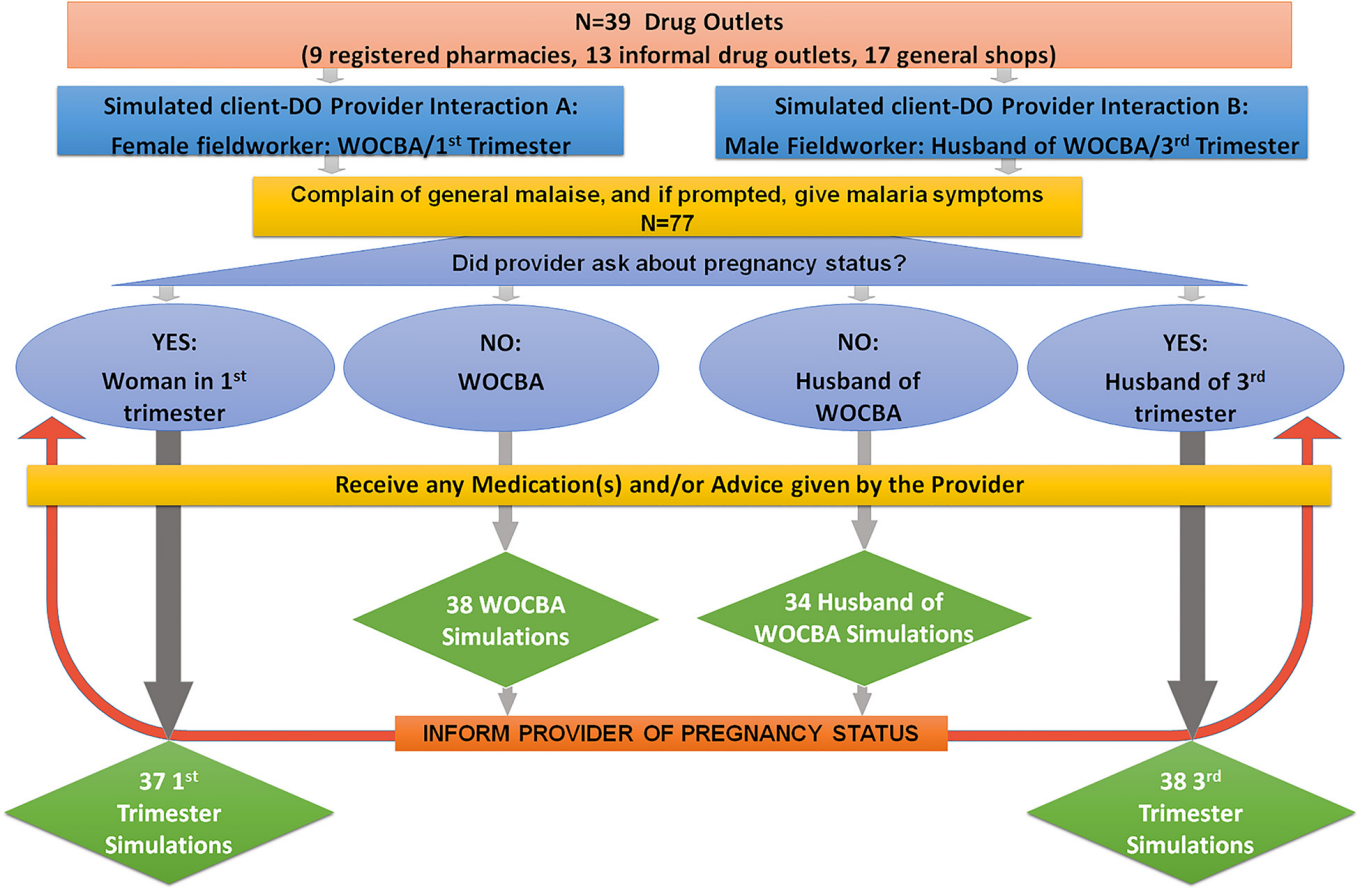
Drug Outlet & Simulation Algorithm.

Twenty-seven home-based shops were excluded from the sampling frame, resulting in 152 consenting DOs. Thirty-nine DOs were then randomly selected (S3 Table). Seventy-seven simulated client-provider encounters with a total of 147 scenarios were completed at 39 DOs (Fig 1). Between 37 and 72 simulations per pregnancy scenario were completed (S3 Table).

#### Pregnancy assessment

DO providers inquired about pregnancy in only 4 of 77 (5%) encounters; pregnancy tests were never offered. DO providers were informed of positive pregnancy status in 70 encounters when there was no initial inquiry on the part of the provider. Pregnancy trimester was assessed in 57% (40/70) of encounters. Inquiry about pregnancy gestation was 77% (27/35) in registered pharmacies and 73% (40/55) in informal drug shops with general shops significantly lower at 31% (20/64), (p<0.01); this did not differ between encounters where the client was the patient versus the patient’s relative (Table 7).

**Table 7:** Pregnancy assessment practice in drug outlets as observed through simulated clients across pregnancy status.

	Overall			WOCBA/1st Trimester			Husband of WOCBA/3rd Trimester			
	N	%	95% CI	N	%	95% CI	N	%	95% CI	P-value
**Pregnancy Inquiry**	77			38			39			
**Unprompted Pregnancy Inquiry**	4	5.2	(3.7, 11.1)	0	0.0		4	10.3	(3.5, 24.2)	0.29
**Confirmation**										
Timing	2	50.0	(0.0, 100.0)	0	0.0		2	50.0	(0.0, 100.0)	
*LMP*	*0*	*0*.*0*		*0*	*0*.*0*		*0*	*0*.*0*		
*Gestation*	*2*	*50*.*0*	*(0*.*0*, *100*.*0)*	*0*	*0*.*0*		*2*	*100*.*0*		
Pregnancy Test Offered	0	0.0		0	0.0		0	0.0		
None	2	50.0	(0.0, 100.0)	0	0.0		2	50.0	(0.0, 100.0)	
**Informed Provider of Pregnancy Status**	70			36			34			0.72
**Confirmation**										
Timing	40	57.1	(45.6, 69.4)	21	58.3	(41.4, 75.2)	19	55.9	(39.0, 72.2)	1.00
*LMP*	*2*	*5*.*0*	*(0*.*0*, *11*.*5)*	*2*	*5*.*6*	*(0*.*0*, *22*.*9)*	*0*	*0*.*0*		
*Gestation*	*38*	*95*.*0*	*(88*.*5*, *100*.*0)*	*19*	*52*.*8*	*(77*.*1*, *100*.*0)*	*19*	*100*.*0*		
Pregnancy Test Offered	0	0.0		0	0.0		0	0.0		
None	30	42.9	(30.6, 54.4)	15	41.7	(24.0, 57.1)	15	44.1	(61.0, 27.8)	1.00
Correct Pregnancy Assessment*	**48**	**62.3**	**(53.1, 65.5)**	**23**	**57.5**	**(48.8, 66.3)**	**25**	**61.0**	**(51.6, 69.4)**	**0.93**

*Correct Pregnancy Assessment indicates that the provider confirmed pregnancy status via LMP, gestational inquiry, or pregnancy test

#### Treatment dispensed

Antimalarials for treatment of malaria were dispensed in 83% (122/147) of all DO encounters; most commonly AL (76%), followed by SP (21%). Quinine was never dispensed (Table 8). There were significant differences in correct treatment across pregnancy status, with 71% (46/72) of non-pregnant, 54% (15/38) of third-trimester, and 0% (0/37) of first-trimester client simulations receiving appropriate treatment (p<0.01). The DO providers were 7.0 times more likely to prescribe SP for treatment of acute malaria to pregnant versus non-pregnant women (95% CI, [2.4–24.9]), p<0.0001). Among providers that were aware the client was in the first trimester, over half (62%) prescribed AL; all but one of the remaining clients were prescribed SP (34%). Artemether-lumefantrine was initially prescribed to over 90% (111/122) of simulated client patients; in 23% (16/70) of encounters in which the provider was informed that the patient was pregnant treatment was changed from AL to SP regardless of trimester, and in another 16% (11/70), AL was withdrawn and the patient was referred to a health facility. Correct case management was observed in only 3% (4/147) of simulations, with no significant difference across outlet types (S7 Table).

**Table 8:** Correct Treatment and Dosage Characteristics by Pregnancy Status in Drug Outlets.

	Overall			WOCBA			1st Trimester			2nd/3rd Trimester			
	N	%	95% CI	N	%	95% CI	N	%	95% CI	N	%	95% CI	P-value
	147			72			37			38			
**Prescribed Antimalarials**	122	**83.0**		65	90.3		29	78.4		28	73.7		
Correct Dosage (tabs x doses x days)*													
Artemether-lumefantrine	93	76.2	(66.3, 86.2)	59	90.8	(81.3, 100.0)	18	62.1	(43.5, 80.6)	16	57.1	(37.9, 76.4)	<0.01
***(4x2x3)***	75	61.5	(51.1, 71.9)	46	70.8	(59.5, 82.0)	14	48.3	(29.1, 67.4)	15	53.6	(34.1, 73.0)	0.04
Artesunate amodiaquine	1	0.8	(0.0, 2.5)	1	1.5	(0.0, 4.7)	0	0.0		0	0.0		
***(4x1x3)***	0	0.0		0	0.0		0	0.0			0.0		
Amodiaquine	2	1.6	(0.0, 5.0)	1	1.5	(0.0, 4.7)	1	3.4	(0.0, 10.4)	0	0.0		
***(3x1x1)***	2	1.6	(0.0, 5.0)	1	1.5		1	3.4	(0.0, 10.4)		0.0		
Quinine	0	0.0		0	0.0		0	0.0		0	0.0		
***(2x3x7)***	0	0.0			0.0			0.0			0.0		
Sulfadoxine Pyrimethamine	26	21.3	(13.1, 29.5)	4	6.2	(0.0, 13.6)	10	34.5	(16.3, 52.7)	12	42.9	(23.6, 62.1)	<0.01
***(3x1x1)***	20	16.4	(8.8, 24.0)	4	6.2	(0.0, 13.6)	6	20.7	(5.2, 36.2)	10	35.7	(17.0, 54.4)	<0.01
Correct Drug	77	63.1		61	93.8		0	0.0		16	57.1		
**Correct Drug & Dosage**	**61**	**50.0**	**(42.5, 57.5)**	**46**	**70.8**	**(59.5, 82.0)**	**0**	**0.0**		**15**	**53.6**	**(34.1, 73.0)**	
**Concomitant Medications**													
Analgesic	90	73.8	(61.2, 86.4)	47	72.3	(59.5, 85.1)	24	82.8	(68.3, 97.2)	19	67.9	(49.7, 86.1)	0.14
Multivitamin	2	1.6	(0.0, 4.8)	0	0.0		0	0.0		2	7.1	(0.0, 17.2)	
**Treatment Advice**													
Dosage directions	106	86.9	(79.2, 94.6)	55	84.6	(68.3, 97.2)	24	82.8	(68.3, 97.2)	27	96.4	(89.2, 100.0)	0.12
Visual Instructions	69	56.6	(43.9, 69.2)	34	52.3	(38.4, 66.2)	18	62.1	(43.5, 80.6)	17	60.7	(41.7, 79.7)	0.47
Emphasize need to finish dose	8	6.6	(0.0, 13.4)	3	4.6	(0.0, 9.9)	1	3.4	(0.0, 10.4)	4	14.3	(0.7, 27.9)	0.01
Side effects	2	1.6	(0.0, 4.0)	1	1.5	(0.0, 4.7)	0	0.0		1	3.6	(0.0, 10.8)	
Advice if symptoms persist	5	4.1	(0.0, 9.0)	3	4.6	(0.0, 9.9)	2	6.9	(0.0, 16.6)	0	0.0		
Any treatment advice	106	86.9	(79.2, 94.6)	55	84.6	(76.2, 93.1)	24	82.8	(68.3, 97.2)	27	96.4	(89.2, 100.0)	0.12

*Percentage for correct dosage is based on the numbers receiving the specific antimalarial

## Discussion

This study found that in Siaya County, Kenya, provider knowledge of the treatment for case management of MiP in both health facilities and drug outlets was poor, with resultant poor levels of observed correct MiP case management practice, particularly in the first trimester of pregnancy (10%). Providers consistently failed to assess for pregnancy, despite the demonstrated knowledge that this step was necessary; practice was considerably worse in DOs than HFs. Although women in second- and third-trimester of pregnancy generally received appropriate therapy, less than one-third of women in first trimester were treated appropriately. Of particular concern, incorrect prescribing practices included provision of AL in early pregnancy, suboptimal dosing of quinine, and use of SP for treatment. These observations highlight the urgent need to monitor and ensure delivery of quality MIP case management.

### Pregnancy Assessment

Pregnancy assessment was very poor. Although 79% of providers reported assessing for pregnancy on the knowledge assessment, less than half of the women in HFs and none of the female simulated clients in DOs were assessed for pregnancy in practice, indicating that providers know they should assess WOCBA for pregnancy but consistently fail to do so. While 52% of HF providers assessed for gestational age, DO providers almost never did so, even when made aware that the woman was pregnant. The failure of providers to assess for pregnancy in a large proportion of women is problematic and may result in inadvertent exposure to potential teratogens, such as ACTs, in early pregnancy. These encounters also represent missed opportunities to refer women to ANC or to remind women of the benefits of early antenatal care in an area where most women initiate ANC late in pregnancy [26].

### Treatment Prescribed

Women in the first trimester were significantly less likely to receive the correct treatment than women in later pregnancy or non-pregnant women. Overall, contraindicated regimens were prescribed in 65% of first-trimester women, consistent with previous observations in this area and neighbouring Uganda [18,27]. This may reflect a reluctance to ask about the culturally sensitive issue of early pregnancy, or a lack of knowledge by healthcare providers regarding potential teratogenicity, as only 56% of providers reported that ACTs were contraindicated in the first trimester. Regardless, provider training should emphasize the potential teratogenicity of ACTs and other potentially harmful drugs, and highlight the importance of identifying pregnant women as early as possible. Five of the providers who were aware that ACTs are contraindicated in the first trimester incorrectly cited SP as the recommended treatment. There was a tendency among DO providers to withdraw AL and refer the woman to a health facility upon learning of pregnancy status. While this may reflect an inadequate knowledge of how to treat pregnant women and may result in delayed receipt of appropriate therapy, referral is preferable to giving an incorrect medication and allows for a complete assessment of the pregnant woman. Creation of clear guidelines for referral to HFs and dissemination of the correct diagnostic and treatment regimens are needed to facilitate correct MiP case management in DOs; only 8% of DOs were in possession of the 2010 MTGs.

Quinine, the recommended treatment in first-trimester pregnancies, was almost never offered, and when it was, dosage was generally incorrect. Poor knowledge of the correct quinine dosage compared to that of other commonly prescribed antimalarials, such as AL, was also observed in the provider survey. At HFs, only 57% of women in first trimester were prescribed quinine, and approximately 60% of those were given an insufficient supply or incorrect instructions. In contrast, only 25% of women given AL received an incorrect dose. These errors resulted in quinine prescriptions ranging from 10–70% of the full dose, increasing the risk of treatment failure and development of drug resistance [28–31].

Quinine was not offered to any of the simulated clients in DOs. This is not surprising given that not a single DO provider cited quinine as the drug of choice for women in the first trimester of pregnancy, suggesting that knowledge, rather than quinine availability or pricing, was the primary issue. However it is notable that quinine (median price = $2.31) was more expensive than AL (median price $0.94) [23]. Inadequate prescribing practices are particularly concerning because quinine is currently the only safe and effective treatment available to women in early pregnancy.

SP was given preferentially to pregnant women in both HFs and DOs; almost 90% of SP prescription in HFs was for treatment of pregnant clients. In many cases in DOs, providers switched therapy from AL to SP after learning that a woman was pregnant. This practice highlights the significant knowledge gap; 49% of DO and 33% of HF providers incorrectly reported that SP could be used for both treatment and prevention. These data raise serious concerns about the dissemination and adoption of treatment guidelines in both the health-facility and drug-outlet sectors, and is particularly alarming in light of the fact that SP has not been recommended as a treatment in Kenya since 2004 [3,10]. Using SP as treatment is associated with a high risk of treatment failure, which could have serious negative health consequences for both the mother and fetus [32–34].

Although other studies have found that availability of drugs and diagnostics, cost, and patient preference all influence provider adherence to case-management guidelines [11,13], in this study, knowledge seemed to be the primary predictor of prescribing practice, particularly in HFs. Most HF had diagnostic tests available [9], all facilities had quinine and AL in stock on the day of the survey, and these drugs are given for free in HFs [23]. Furthermore, no HF provider cited patient preference as a reason for prescribing medicines during the knowledge assessment interviews. In DOs, the situation was a bit different: diagnostic capacity was limited [9], only 34% of DOs in the HDSS had quinine in stock at the time of the survey [23], quinine was more expensive than AL (median prices $2.31 and $0.94, respectively) [23], and 8% of providers did cite drug availability and patient preference as a factor influencing drug choice. This may reflect the differences in the clients seeking care from HF versus DOs- previous studies have found that patients concerned about affordability, access, and acquiring their drug of preference are more likely to seek treatment from drug outlets [35].

### Improving Provider Knowledge of and Adherence to the National Malaria Treatment Guidelines

Health-facility providers had significantly greater knowledge of MiP consequences, clinical symptoms, pregnancy assessment, and treatment regimens versus DO providers. However, the only significant indicators for correct knowledge were malaria management training and professional cadre, which was reflected in the differences by facility type, consistent with previous findings [13]. Training alone has been shown to have limited impact on provider case management practice [13]. Although this study was conducted in the HDSS area, the study did not make use of the HDSS structures, and facilities with ongoing studies were excluded. Therefore, these results are generalizable to the non-HDSS setting in Kenya, and likely to other countries in sub-Saharan Africa.

A different combination of approaches and interventions are likely to work for HFs versus DOs. Knowledge survey results showed that media plays an important role as source of information for both HF and DO providers and should be explored as a potential platform for providing health messages. The use of mobile phone text-message reminders (mHealth) has been shown to improve malaria case management practice for children in Kenya, and could be combined with simple, focused training to improve case management in pregnancy [36]. Team-based quality improvement has been suggested as another method to improve provider practice [37]. Whether DOs should strictly refer, or can also actively manage MIP cases, is likely to require policy clarification, particularly with respect to each DO category. The role and place of registered pharmacies in the health system and market can vary significantly from other DOs that are not registered with an official regulatory body. Updated guidance for all entities must be disseminated along with targeted MiP trainings and point-of-service job aids with increased adherence to Ministry of Health supportive supervision guidelines [13,37,38]. New government policy that regulates and provides clear guidelines for registered drug outlet practice is needed in light of the dismal performance observed. Governmental recognition of informal drug outlets, under the recommended regulation and guidance, may also be relevant given patient health-seeking behaviours. A registration system for informal DOs, similar to that of the accredited drug-dispensing outlets in Tanzania, may increase competition with registered pharmacies and incentivize both entities to improve their practice through public recognition of DO entities with higher knowledge levels [39].

Improvement of pregnancy assessment is needed but will be a challenge due to socio-cultural factors that influence both a woman’s willingness to disclose pregnancy status and a provider’s willingness to ask. Adequate guidelines for pregnancy assessment of all WOCBA and interactive trainings must be available so that providers feel comfortable inquiring about potential pregnancy. Providers must clearly explain the purpose of the pregnancy assessment (i.e., to ensure adequate and safe treatment) and be prepared to refer the patient for ANC services [40]. In addition, a community outreach component to educate women on the importance of sharing pregnancy status with healthcare providers will likely be needed to facilitate the patient/ provider interaction. Multiple coordinated approaches and overall capacity building will be important keys to the improvement of MiP case management practice across facilities and has been shown to be effective in the region [37,38].

### Limitations & Challenges

The relatively short time-frame of the study limited the number of exit interviews. In particular, the identification of febrile patients in first trimester was challenging, probably due to limitations in early pregnancy detection. Gestational age assessment was based on reported LMP, which could have led to misclassification of pregnancy trimester for late first trimester pregnancies. However, unless the provider used an alternative approach to assess gestation (e.g., fundal height) it is unlikely that assessment of correct practice would have been affected.

Exit interviews and provider surveys were susceptible to courtesy or social-desirability bias, meaning that respondents may have provided answers they thought were ‘more correct’ or that the interviewer wanted to hear. Data obtained from exit interviews might be biased due to patient recall or information loss, although the interview was conducted immediately upon completion of the consultation to minimize this factor. In addition, errors may have been introduced if the patient did not understand the information given or procedures completed by the provider. Finally, in the health facility exit interviews, we did not record whether patients had been seen in the ANC or the OPD, nor whether the woman had been seen in the ANC earlier during the pregnancy. This limits our ability to assess whether pregnancy testing was needed for a particular woman, as well as to identify where to target additional training.

## Conclusion

We observed very poor case management practice and knowledge for malaria in pregnancy in both HFs and DOs in Siaya County, western Kenya. Particularly concerning were the failure of providers to assess WOCBA for pregnancy status, incorrect treatment with SP, inadequate quinine dosage, and AL prescribed in first-trimester pregnancies. Multifaceted approaches, including focused trainings, mHealth, team-based quality improvement, and supportive supervision should be implemented to improve provider adherence and knowledge. These approaches should be tailored specifically for HFs and DOs given the unique provider qualifications and patient health-seeking behaviours that characterize the two entities. Improving practice in the informal sector is critical, as it comprises a large part of health service provision for malaria treatment and has little-to-no regulatory oversight. Optimizing treatment of WOCBA and pregnant women is critical to prevent adverse consequences of MiP.

## Disclaimer

The findings and conclusions presented in this manuscript are those of the authors and do not necessarily reflect the official position of the U.S. President’s Malaria Initiative, U.S. Agency for International Development, or U.S. Centers for Disease Control and Prevention.

## Supporting Information

S1 Table Malaria Treatment Guideline Awareness, comparing Health Facilities vs. Drug Outlets. (DOCX)

S2 Table Reasons for Malaria Treatment & Contraindications among Health Facilities and Drug Outlets. (DOCX)

S3 Table Facility Characteristics by Health Facilities and Drug Outlets. (DOCX)

S4 Table Health Facility Exit Interview: Respondent Characteristic. (DOCX)

S5 Table Malaria Case Management Practice in Health Facilities as observed through Exit Interviews, stratified across Health Facility Type. (DOCX)

S6 Table Provider Characteristic Predictors of correct prescribing and diagnostic practice in health facilities. (DOCX)

S7 Table Malaria Case Management practice in drug outlets as observed through simulated clients stratified across Drug Outlet Type. (DOCX)

S8 Table Comprehensive Care Practices Provided during Pregnancy, comparing Health Facilities vs. Drug Outlets. (DOCX)
