# Supplementary material for: Knowledge and Adherence to the National Guidelines for Malaria Case Management in Pregnancy among Healthcare Providers and Drug Outlet Dispensers in Rural, Western Kenya
Source: PLoS One. 2016 Jan 20;11(1):e0145616. doi: 10.1371/journal.pone.0145616 (PMC4720358; doi:10.1371/journal.pone.0145616)
Supplement: S1 Table — (DOCX) [file pone.0145616.s001.docx]

**Table S1. Malaria Treatment Guideline Awareness, comparing Health Facilities vs. Drug Outlets**

|  | Total | | | Health Facilities | | | Drug Outlets | | |  |
| --- | --- | --- | --- | --- | --- | --- | --- | --- | --- | --- |
| **MTGs** | n=112 | % | 95% CI | n=75 | % | 95% CI | n=37 | % | 95%CI | P-value |
| Awareness of Government Initiative | 65 | 58.0 | (47.7, 68.4) | 62 | 82.7 | (72.6, 92.7) | 3 | 8.1 | (0.0, 17.1) | <0.01 |
| Read the MTGs | 75 | 67.0 | (57.3, 76.6) | 67 | 89.3 | (82.3, 96.4) | 8 | 21.6 | (8.1, 35.2) | <0.01 |
| In Possession | 63 | 56.3 | (45.9, 66.6) | 60 | 80.0 | (70.1, 89.9) | 3 | 8.1 | (0.0, 17.1) | <0.01 |
| Additional Materials | 73 | 65.2 | (55.2, 75.1) | 71 | 94.7 | (89.6, 99.8) | 2 | 5.4 | (0.0, 12.8) | <0.01 |
| **Awareness of MTGs** | **84** | **75.0** | **(66.1, 83.9)** | **74** | **98.7** | **(96.0, 100.0)** | **10** | **27.0** | **(12.4, 41.6)** | <0.01 |
| **Addtl Sources of Information** |  |  |  |  |  |  |  |  |  |  |
| Training/CME | 63 | 56.3 | (46.4, 66.1) | 52 | 69.3 | (57.9, 80.8) | 11 | 29.7 | (14.7, 44.8) | <0.01 |
| DHMT/health facility memos | 46 | 41.1 | (31.5, 50.7) | 38 | 50.7 | (38.8, 62.5) | 8 | 21.6 | (8.1, 35.2) | <0.01 |
| Colleagues | 40 | 35.7 | (26.0, 45.4) | 28 | 37.3 | (25.1, 49.6) | 12 | 32.4 | (17.0, 47.8) | 0.6215 |
| Media | 55 | 49.1 | (38.7, 59.5) | 33 | 44.0 | (30.9, 57.1) | 22 | 59.5 | (43.3, 75.6) | 0.1405 |
| Medical Journals | 19 | 17.0 | (8.6, 25.3) | 19 | 25.3 | (13.2, 37.5) | 0 | 0.0 |  |  |
| Medical Reps | 17 | 15.2 | (8.2, 22.1) | 11 | 14.7 | (6.2, 23.1) | 6 | 16.2 | (4.1, 28.3) | 0.8322 |
| Other* | 10 | 8.9 | (3.5, 14.4) | 3 | 4.0 | (0.0, 8.5) | 7 | 18.9 | (6.0, 31.8) | <0.01 |
| **Training Workshops** |  |  |  |  |  |  |  |  |  |  |
| Malaria Training | 61 | 54.5 | (45.2, 63.7) | 51 | 68.0 | (58.0, 78.0) | 10 | 27.0 | (12.4, 41.6) | <0.01 |
| *within past 5 years* | 57 | 50.9 | (41.3, 60.5) | 50 | 66.7 | (56.1, 77.3) | 7 | 18.9 | (6.0, 31.8) | <0.01 |
| MIP Training | 34 | 30.4 | (22.4, 38.3) | 30 | 40.0 | (30.2, 49.8) | 4 | 10.8 | (0.6, 21.0) | <0.01 |
| *within past 5 years* | 31 | 27.7 | (19.9, 35.5) | 28 | 37.3 | (27.3, 47.3) | 3 | 8.1 | (0.0, 17.1) | <0.01 |

### **P-values from Chi-square test*

### ***Other includes community meetings (Barazas), CDC staff, NGOs, & Village Reporters*

### *Acronyms: MTGs, malaria treatment guidelines; CME, continuing medical education; DHMT, district health medical team; MiP, malaria in pregnancy*
