# Supplementary material for: Knowledge and Adherence to the National Guidelines for Malaria Case Management in Pregnancy among Healthcare Providers and Drug Outlet Dispensers in Rural, Western Kenya
Source: PLoS One. 2016 Jan 20;11(1):e0145616. doi: 10.1371/journal.pone.0145616 (PMC4720358; doi:10.1371/journal.pone.0145616)
Supplement: S2 Table — (DOCX) [file pone.0145616.s002.docx]

Table S2. Reasons for Malaria Treatment & Contraindications among Health Facilities and Drug Outlets

|  |  |  | **Overall** | | | **Health Facilities** | | | **Drug Outlets** | | |
| --- | --- | --- | --- | --- | --- | --- | --- | --- | --- | --- | --- |
|  |  |  | **n=112** | **%** | **95% CI** | **n=75** | **%** | **95% CI** | **n=37** | **%** | **95% CI** |
| **1st Line Treatment Reasoning** | | |  |  |  |  |  |  |  |  |  |
| Due to National Guidelines | | | 39 | 34.8 | (26.2, 43.4) | 30 | 40.0 | (29.4, 50.6) | 9 | 24.3 | (10.2, 38.4) |
| Found Effective in Practice | | | 51 | 45.5 | (35.9, 55.2) | 35 | 46.7 | (34.8, 58.5) | 16 | 43.2 | (27.0, 59.5) |
| Affordable/Cheap | |  | 9 | 8.0 | (3.0, 13.1) | 7 | 9.3 | (2.8, 15.8) | 2 | 5.4 | (0.0, 12.8) |
| Safe |  |  | 3 | 2.7 | (0.0, 5.7) | 1 | 1.3 | (0.0, 4.0) | 2 | 5.4 | (0.0, 12.8) |
| Don’t Know | |  | 7 | 6.3 | (1.7, 10.8) | 2 | 2.7 | (0.0, 6.3) | 5 | 13.5 | (2.3, 24.8) |
| Other |  |  | 3 | 2.7 | (0.0, 5.7) | 0 | 0.0 |  | 3 | 8.1 | (0.0, 17.1) |
| **Other included availability & patient choice* | | | |  |  |  |  |  |  |  |  |
| **2nd Line Treatment Reasoning** | | |  |  |  |  |  |  |  |  |  |
| Due to National Guidelines | | | 34 | 30.4 | (21.7, 39.0) | 27 | 36.0 | (24.9, 47.1) | 7 | 18.9 | (6.0, 31.8) |
| Found Effective in Practice | | | 41 | 36.6 | (27.4, 45.9) | 32 | 42.7 | (31.1, 54.3) | 9 | 24.3 | (10.2, 38.4) |
| Affordable/Cheap | |  | 5 | 4.5 | (0.6, 8.3) | 5 | 6.7 | (0.9, 12.4) | 0 | 0.0 |  |
| Safe |  |  | 2 | 1.8 | (0.0, 4.2) | 2 | 2.7 | (0.0, 6.2) | 0 | 0.0 |  |
| Don’t Know | |  | 23 | 20.5 | (12.8, 28.3) | 5 | 6.7 | (1.1, 12.2) | 18 | 48.6 | (32.2, 65.1) |
| Other |  |  | 7 | 6.3 | (1.8, 10.7) | 4 | 5.3 | (0.4, 10.3) | 3 | 8.1 | (0.0, 17.1) |
| **Other included resistance/treatment failure* | | | |  |  |  |  |  |  |  |  |
| **ACT Contraindications** | | | **n=95** | **%** | **95% CI** | **n=69** | **%** | **95% CI** | **n=26** | **%** | **95% CI** |
| Trimesters: | | 1st | 60 | 63.2 | (52.9, 73.5) | 53 | 76.8 | (66.5, 87.1) | 7 | 26.9 | (9.5, 44.4) |
|  |  | 2nd | 1 | 1.1 | (0.0, 3.2) | 0 | 0.0 |  | 1 | 3.8 | (0.0, 11.4) |
|  |  | 3rd | 1 | 1.1 | (0.0, 3.1) | 1 | 1.4 | (0.0, 4.2) | 0 | 0.0 |  |
|  |  | All | 6 | 6.3 | (1.3, 11.4) | 1 | 1.4 | (0.0, 4.3) | 5 | 19.2 | (3.7, 34.7) |
| Lactating Mothers | |  | 4 | 4.2 | (0.1, 8.4) | 4 | 5.8 | (0.1, 11.5) | 0 | 0.0 |  |
| Allergy |  |  | 27 | 28.4 | (18.9, 38.0) | 24 | 34.8 | (22.9, 46.7) | 3 | 11.5 | (0.0, 24.1) |
| None |  |  | 7 | 7.4 | (1.9, 12.9) | 3 | 4.3 | (0.0, 9.4) | 4 | 15.4 | (1.2, 29.6) |
| Don't Know | |  | 5 | 5.3 | (0.6, 9.9) | 1 | 1.4 | (0.0, 4.4) | 4 | 15.4 | (1.2, 29.6) |

*Of the providers that cited the national guidelines as the reason, 5% and 68% had chosen an antimalarial not recommended by the national MTGs for 1^st^-line and 2^nd^-line, respectively*
