# Supplementary material for: Knowledge and Adherence to the National Guidelines for Malaria Case Management in Pregnancy among Healthcare Providers and Drug Outlet Dispensers in Rural, Western Kenya
Source: PLoS One. 2016 Jan 20;11(1):e0145616. doi: 10.1371/journal.pone.0145616 (PMC4720358; doi:10.1371/journal.pone.0145616)
Supplement: S3 Table — (DOCX) [file pone.0145616.s003.docx]

Table S3. Facility Characteristics by Health Facilities and Drug Outlets

| **Facility Characteristics** | ***Health Facilities*** | | ***Drug Outlets*** | |
| --- | --- | --- | --- | --- |
|  | **N=51** | **%** | **N=39** | **%** |
| **Facility Managing Authority** |  |  |  |  |
| Government | 44 | 86.3 | 0 | 0.0 |
| Mission | 2 | 3.9 | 0 | 0.0 |
| Private | 5 | 9.8 | 39 | 100.0 |
| **Facility Type** |  |  |  |  |
| Hospital | 4 | 7.8 | -- |  |
| Health Center | 19 | 37.3 | -- |  |
| Dispensary | 28 | 54.9 | -- |  |
| **Total Health Facilities** | **51** | **100.0** | **--** |  |
| Registered Pharmacy | -- |  | 9 | 23.1 |
| Informal Drug Shop | -- |  | 13 | 33.3 |
| General Shop | -- |  | 17 | 43.6 |
| **Total Drug Outlets** | -- |  | **39** | **100.0** |
| **Patient Encounter Method** | ***Patient Interviews*** | | ***Simulated Clients*** | |
|  | ***N=208*** | ***%*** | ***N=147*** | ***%*** |
| **Pregnancy Status** |  |  |  |  |
| Non-pregnant | 111 | 53.4 | 72 | 49.3 |
| 1st Trimester | 21 | 10.1 | 37 | 25.3 |
| 2nd/3rd Trimester | 76 | 36.5 | 38 | 26.0 |
