# Supplementary material for: Knowledge and Adherence to the National Guidelines for Malaria Case Management in Pregnancy among Healthcare Providers and Drug Outlet Dispensers in Rural, Western Kenya
Source: PLoS One. 2016 Jan 20;11(1):e0145616. doi: 10.1371/journal.pone.0145616 (PMC4720358; doi:10.1371/journal.pone.0145616)
Supplement: S4 Table — (DOCX) [file pone.0145616.s004.docx]

Table S4. Health Facility Exit Interview: Respondent Characteristic

|  | ***Overall*** | | | ***Non-Pregnant*** | | | ***1st trimester***** | | ***2nd/3rd trimester*** | | |
| --- | --- | --- | --- | --- | --- | --- | --- | --- | --- | --- | --- |
|  | ***N=208*** | | ***%*** | ***N=111*** | ***%*** | | ***N=21*** | ***%*** | ***N=76*** | | ***%*** |
| **Respondent Characteristics** | | |  |  |  | |  |  |  | |  |
| **Age mean *(range) &* Std. Deviation** | | **26.4 *(17-48)*** | **7.2** | **28.2 *(18-48)*** | **7.8** | | **25.6 *(18-45)*** | **6.5** | **23.9 *(17-40)*** | | **5.6** |
| **Education Level** | |  |  |  |  | |  |  |  | |  |
| No Education | | 16 | 7.7 | 14 | 12.6 | | 1 | 4.8 | 1 | | 1.3 |
| Primary | | 137 | 65.9 | 70 | 63.1 | | 15 | 71.4 | 52 | | 68.4 |
| Secondary | | 38 | 18.3 | 20 | 18.0 | | 2 | 9.5 | 16 | | 21.1 |
| Higher Education | | 17 | 8.2 | 7 | 6.3 | | 3 | 14.3 | 7 | | 9.2 |
| **Symptoms Reported to provider*** | | | | | | | | | | | |
| Fever | 137 | | 65.9 | 78 | | 70.3 | 14 | 66.7 | | 45 | 59.2 |
| Headache | 182 | | 87.5 | 101 | | 91.0 | 18 | 85.7 | | 63 | 82.9 |
| Pain | 104 | | 50.0 | 54 | | 48.6 | 10 | 47.6 | | 24 | 31.6 |
| Nausea | 72 | | 34.6 | 27 | | 24.3 | 7 | 33.3 | | 31 | 40.8 |
| Malaise | 79 | | 38.0 | 39 | | 35.1 | 6 | 28.6 | | 34 | 44.7 |
| Chills | 17 | | 8.2 | 9 | | 8.1 | 2 | 9.5 | | 6 | 7.9 |
| Stomach Pain | 23 | | 11.1 | 12 | | 10.8 | 3 | 14.3 | | 8 | 10.5 |
| Cough | 18 | | 8.7 | 7 | | 6.3 | 3 | 14.3 | | 8 | 10.5 |
| Dizziness | 3 | | 1.4 | 0 | | 0.0 | 0 | 0.0 | | 3 | 3.9 |
| Diarrhea | 2 | | 1.0 | 0 | | 0.0 | 1 | 4.8 | | 1 | 1.3 |
| **Gravidity** | n=156 | |  | n=64 | |  | n=20 |  | | n=71 |  |
| 0 | 33 | | 21.2 | 12 | | 18.8 | 3 | 15.0 | | 18 | 25.4 |
| 1 | 31 | | 19.9 | 11 | | 17.2 | 6 | 30.0 | | 13 | 18.3 |
| 2 | 33 | | 21.2 | 12 | | 18.8 | 3 | 15.0 | | 18 | 25.4 |
| 3-4 | 32 | | 20.5 | 12 | | 18.8 | 6 | 30.0 | | 14 | 19.7 |
| 5+ | 27 | | 17.3 | 17 | | 26.6 | 2 | 10.0 | | 8 | 11.27 |
| ***Missing*** | ***53*** | | **25.4** | ***47*** | | **42.3** | ***1*** | **4.8** | | ***5*** | **6.6** |

**2 reported no symptoms to provider*

***Patients with gestational age of up to 14 weeks, 6 days were included in 1st trimester given that treatment guidelines use 'quickening' as a treatment indicator*
