# Supplementary material for: Knowledge and Adherence to the National Guidelines for Malaria Case Management in Pregnancy among Healthcare Providers and Drug Outlet Dispensers in Rural, Western Kenya
Source: PLoS One. 2016 Jan 20;11(1):e0145616. doi: 10.1371/journal.pone.0145616 (PMC4720358; doi:10.1371/journal.pone.0145616)
Supplement: S5 Table — (DOCX) [file pone.0145616.s005.docx]

Table S5. Malaria Case Management Practice in Health Facilities as observed through exit interviews, stratified across Health Facility Type

|  | ***Overall*** | | | **Hospital** | | | **Health Center** | | | **Dispensary** | | |  |
| --- | --- | --- | --- | --- | --- | --- | --- | --- | --- | --- | --- | --- | --- |
|  | ***N*** | ***%*** | ***95% CI*** | ***N*** | ***%*** | ***95% CI*** | ***N*** | ***%*** | ***95% CI*** | ***N*** | ***%*** | ***95% CI*** | ***P-value*** |
|  | 208 |  |  | 18 |  |  | 83 |  |  | 107 |  |  |  |
| **Malaria Diagnostics** | 187 | 89.9 | (85.1, 94.7) | 16 | 88.9 | (68.2, 100.0) | 79 | 95.2 | (90.4, 99.9) | 92 | 86.0 | (79.0, 93.0) | 0.20 |
| **Pregnancy Assessment** | 107 | 51.4 | (41.9, 61.0) | 14 | 77.8 | (62.3, 93.2) | 45 | 54.2 | (33.9, 74.5) | 48 | 44.9 | (36.0, 53.8) | 0.10 |
| **Treatment & Dosage*** | 129 | 62.0 | (52.2, 71.8) | 10 | 55.6 | (19.2, 91.9) | 54 | 65.1 | (49.9, 80.2) | 65 | 60.7 | (47.1, 74.4) | 0.85 |
| Non-pregnant *n=111* | 76 | 68.5 | (57.2, 79.7) | 7 | 77.8 | (49.6, 100.0) | 31 | 72.1 | (57.5, 83.4) | 38 | 67.9 | (47.2, 83.6) | 0.75 |
| 1st Trimester *n=22* | 5 | 23.8 | (4.0, 43.6) | 0 | 0.0 |  | 3 | 60.0 | (0.0, 87.3) | 2 | 16.7 | (0.0, 38.4) |  |
| 2nd/3rd Trimester *n=76* | 49 | 64.5 | (49.5, 76.8) | 3 | 42.9 | (0.0, 86.7) | 20 | 62.5 | (39.1, 85.9) | 26 | 68.4 | (50.4, 84.8) | 0.58 |
| **Correct Practice** | **65** | **31.3** | **(21.9, 40.6)** | **7** | **38.9** | **(13.9, 63.9)** | **29** | **34.9** | **(15.5, 54.4)** | **29** | **27.1** | **(17.5, 36.7)** | **0.61** |

**All facilities had both quinine and artemether-lumefantrine in stock on the day the exit interviews were conducted*
