# Supplementary material for: Knowledge and Adherence to the National Guidelines for Malaria Case Management in Pregnancy among Healthcare Providers and Drug Outlet Dispensers in Rural, Western Kenya
Source: PLoS One. 2016 Jan 20;11(1):e0145616. doi: 10.1371/journal.pone.0145616 (PMC4720358; doi:10.1371/journal.pone.0145616)
Supplement: S6 Table — (DOCX) [file pone.0145616.s006.docx]

**Table S6. Provider characteristic predictors related to correct prescribing practice in health facilities**

| **Provider Characteristic** | **N** | % | **Crude OR** | **95% CI** | **Pr >\|Z\|** | **Adjusted OR** | **95% CI** | **Pr >\|Z\|** |
| --- | --- | --- | --- | --- | --- | --- | --- | --- |
| **Respondent cadre** | **184** |  |  |  |  |  |  |  |
| Nurse (ref) | 109 | 59.2 | -- | -- | -- | -- | -- | -- |
| Clinical Officer/M.D. | 52 | 28.3 | 1.5 | (0.6, 3.7) | 0.39 | 1.6 | (0.6, 3.9) | 0.34 |
| Pharmacist | 5 | 2.7 | 8.8 | (1.0, 78.5) | 0.05 | 8.2 | (0.9, 74.1) | 0.06 |
| Other | 18 | 9.8 | <0.01 | (<0.01, <0.01) | <0.001 | <0.01 | (<0.01, <0.01) | <0.001 |
| **Dispenses medicine (ref='No')** |  |  |  |  |  |  |  |  |
| No | 19 | 10.3 | -- | -- | -- | -- | -- | -- |
| yes | 165 | 89.7 | 1.9 | (1.0, 3.6) | 0.05 | 2.2 | (1.2, 4.3) | 0.02 |
