# Supplementary material for: Knowledge and Adherence to the National Guidelines for Malaria Case Management in Pregnancy among Healthcare Providers and Drug Outlet Dispensers in Rural, Western Kenya
Source: PLoS One. 2016 Jan 20;11(1):e0145616. doi: 10.1371/journal.pone.0145616 (PMC4720358; doi:10.1371/journal.pone.0145616)
Supplement: S7 Table — (DOCX) [file pone.0145616.s007.docx]

Table S7. Malaria Case Management practice in drug outlets as observed through simulated clients stratified across Drug Outlet Type

|  | ***Overall*** | | | **Registered Pharmacy** | | | **Informal Drug Shop** | | | **General Shop** | | |  |
| --- | --- | --- | --- | --- | --- | --- | --- | --- | --- | --- | --- | --- | --- |
|  | ***N*** | ***%*** | ***95% CI*** | ***N*** | ***%*** | ***95% CI*** | ***N*** | ***%*** | ***95% CI*** | ***N*** | ***%*** | ***95% CI*** | ***P-value*** |
|  | 147 |  |  | 35 |  |  | 48 |  |  | 64 |  |  |  |
| **Malaria Diagnostics** | 48 | 32.7 | (21.5, 43.8) | 13 | 37.1 | (11.3, 63.0) | 21 | 43.8 | (22.6, 64.9) | 14 | 21.9 | (9.3, 34.5) | 0.20 |
| **Pregnancy Assessment** | 43 | 29.3 | (23.0, 35.5) | 14 | 40 | (30.3, 49.7) | 18 | 37.5 | (27.2, 47.8) | 11 | 17.2 | (9.8, 24.5) | <0.01 |
| **Treatment & Dosage** | 61 | 41.5 | (33.2, 49.8) | 18 | 51.4 | (42.7, 60.2) | 25 | 52.1 | (39.7, 64.4) | 18 | 28.1 | (14.8, 41.4) | <0.01 |
| Non-pregnant *n=72* | 46 | 63.9 | (51.6, 76.2) | 14 | 82.4 | (65.7, 99.0) | 19 | 82.6 | (67.5, 97.7) | 13 | 40.6 | (22.4, 58.9) | <0.01 |
| 1st Trimester *n=37* | 0 | 0.0 |  | 0 | 0.0 |  | 0 | 0.0 |  | 0 | 0.0 |  |  |
| 2nd/3rd Trimester *n=38* | 15 | 39.5 | (23.2, 55.8) | 4 | 44.4 | (10.4, 78.5) | 6 | 46.2 | (17.8, 74.5) | 5 | 31.3 | (7.5, 55.0) | 0.67 |
| **Correct Practice** | **4** | **2.7** | **(0.1, 5.4)** | **1** | **2.9** | **(0.0, 8.5)** | **1** | **2.1** | **(0.0, 6.3)** | **2** | **3.1** | **(0.0, 7.4)** | **0.94** |
