# Supplementary material for: Knowledge and Adherence to the National Guidelines for Malaria Case Management in Pregnancy among Healthcare Providers and Drug Outlet Dispensers in Rural, Western Kenya
Source: PLoS One. 2016 Jan 20;11(1):e0145616. doi: 10.1371/journal.pone.0145616 (PMC4720358; doi:10.1371/journal.pone.0145616)
Supplement: S8 Table — (DOCX) [file pone.0145616.s008.docx]

Table S8. Comprehensive Care Practices Provided during Pregnancy, comparing Health Facilities vs. Drug Outlets

|  |  |  | **Overall** | | | **Health Facilities** | | | **Drug Outlets** | | |  |
| --- | --- | --- | --- | --- | --- | --- | --- | --- | --- | --- | --- | --- |
|  |  |  | **n=112** | **%** | **95% CI** | **n=75** | **%** | **95% CI** | **n=37** | **%** | **95% CI** | **p-value^#^** |
| **Pregnant patients given information with treatment** | | |  |  |  |  |  |  |  |  |  |  |
| Instructions | |  | 97 | 86.6 | (80.2, 93.0) | 71 | 94.7 | (89.7, 99.6) | 26 | 70.3 | (55.2, 85.3) | <0.01 |
| Side Effects | |  | 64 | 57.1 | (46.4, 67.9) | 57 | 76.0 | (64.0, 88.0) | 7 | 18.9 | (6.0, 31.8) | <0.01 |
| Return if Symptoms Continue | | | 58 | 51.8 | 41.4, 62.2) | 47 | 62.7 | (49.3, 76.0) | 11 | 29.7 | (14.7, 44.8) | <0.01 |
| Danger Signs** | |  | 8 | 7.1 | (2.4, 11.9) | 5 | 6.7 | (1.1, 12.3) | 3 | 8.1 | (0.0, 17.1) | 0.78 |
| Other |  |  | 12 | 10.7 | (4.7, 16.7) | 3 | 4.0 | (0.0, 8.5) | 9 | 24.3 | (10.2, 38.4) | <0.01 |
| **Any Information Given** | |  | **102** | **91.1** | **(85.5, 96.6)** | **74** | **98.7** | **(96.0, 100.0)** | **28** | **75.7** | **(61.6, 89.8)** | <0.01 |
| **Care practices in pregnancy^€^** | | |  |  |  |  |  |  |  |  |  |  |
| Prevent Hypoglycemia | |  | 32 | 28.6 | (18.4, 38.7) | 32 | 42.7 | (29.2, 56.1) | 0 | 0.0 |  |  |
| Fetal Monitoring | |  | 50 | 44.6 | (34.2, 55.1) | 45 | 60.0 | (46.9, 73.1) | 5 | 13.5 | (2.3, 24.8) |  |
| Anemia Treatment | |  | 50 | 44.6 | (34.8, 54.5) | 46 | 61.3 | (49.5, 73.2) | 4 | 10.8 | (0.6, 21.0) |  |
| Antipyretics | |  | 25 | 22.3 | (14.2, 30.5) | 24 | 32.0 | (21.1, 42.9) | 1 | 2.7 | (0.0, 8.0) |  |
| None |  |  | 8 | 7.1 | (2.2, 12.1) | 0 | 0.0 |  | 8 | 21.6 | (8.1, 35.2) |  |
| Other* |  |  | 53 | 47.3 | (38.5, 57.2) | 25 | 33.3 | (21.9, 44.8) | 28 | 75.7 | (61.6, 89.8) |  |

*€ Provision of clinical care is not within the scope of a drug outlet, thus no comparison was made between health facilities and drug outlets.*

**Included nutritious diet, ITNs, IPTp, and medication compliance.*

*** Danger signs included death, convulsions, dizziness, spotting, & fetal movement*

*# P-values from Chi-square test and Fisher Exact used for strata with <5 observations*
